# Scuttling in the highlands: Discovery of a new genus and species of freshwater crabs (Decapoda, Gecarcinucidae) from the Eastern Ghats, India

**DOI:** 10.3897/zookeys.1257.156494

**Published:** 2025-10-28

**Authors:** Santanu Mitra, Shibananda Rath, Won-Kyo Jung, Hyun-Woo Kim, Shantanu Kundu

**Affiliations:** 1 Crustacea Division, Zoological Survey of India, Fire Proof Spirit Building, 27 Jawaharlal Nehru Road, Kolkata 700016, India Zoological Survey of India Kolkata India; 2 Freshwater Fish Section, Zoological Survey of India, Fire Proof Spirit Building, 27 Jawaharlal Nehru Road, Kolkata 700016, India Pukyong National University Busan Republic of Korea; 3 Marine Integrated Biomedical Technology Center, National Key Research Institutes in Universities, Pukyong National University, Busan 48513, Republic of Korea Zoological Survey of India Kolkata India; 4 Research Center for Marine Integrated Bionics Technology, Pukyong National University, Busan 48513, Republic of Korea Pukyong National University Busan Republic of Korea; 5 Major of Biomedical Engineering, Division of Smart Healthcare and New-Senior Healthcare Innovation Center (BK21 Plus), Pukyong National University, Busan, 48513, Republic of Korea Zoological Survey of India Kolkata India; 6 Department of Marine Biology, Pukyong National University, Busan 48513, Republic of Korea Pukyong National University Busan Republic of Korea; 7 Ocean and Fisheries Development International Cooperation Institute, College of Fisheries Science, Pukyong National University, Busan 48513, Republic of Korea Zoological Survey of India Kolkata India; 8 International Graduate Program of Fisheries Science, Pukyong National University, Busan 48513, Republic of Korea Pukyong National University Busan Republic of Korea

**Keywords:** Crustaceans, Eastern Ghat, endemic species, integrated approach, new genus, new species, taxonomy

## Abstract

A new genus of gecarcinucid freshwater crab, *Patithelphusa***gen. nov.**, is described from the Shevaroy Hills, Yercaud, located in Salem District, Tamil Nadu, within the Eastern Ghats of India. This new genus is closely related to *Baratha* Bahir & Yeo, 2007 and *Travancoriana* Bott, 1969, but can be readily distinguished by a combination of morphological characters, including a relatively broad and shallow cervical groove, triangular bilobed median teeth on the epistomal median lobe, a blunt external orbital tooth with a relatively long outer margin, and a distinct G1 terminal segment terminating in a triangular tip. In addition, morphological characters together with molecular evidence from partial mitochondrial 16S rRNA gene sequences support the recognition of *Patithelphusa yercaudensis***sp. nov.** as a distinct species, exhibiting a genetic divergence of 9.66% from its closest relative, *Travancoriana schirnerae* Bott, 1969. Phylogenetic analyses based on Bayesian inference and maximum-likelihood approaches recovered the newly described species as a well-supported monophyletic clade, distinctly separated from other known gecarcinucid taxa from India and the geographically proximate region of Sri Lanka. With the addition of this new genus and species, the diversity of Indian gecarcinucid crabs increases to 112 species across 31 genera. Furthermore, biogeographic inferences suggest that the new genus and species may be isolated from its closest gecarcinucid relatives by the wide valleys and the Cauvery River system, which likely act as ecological and geographical barriers. These unique topographical features may restrict gene flow and limit dispersal among high-altitude, habitat-specialist decapods, providing a refuge that facilitates the evolution of novel taxonomic units in peninsular India.

## ﻿Introduction

The Indian subcontinent harbours a rich diversity of freshwater crabs (Crustacea: Decapoda), comprising approximately 172 species, which account for around 10% of the global brachyuran diversity (Pati et al. 2023; Mitra and Khamo 2024). Notably, the families Potamidae and Gecarcinucidae exhibit the highest species richness within this region (Pati and Pradhan 2020). Over the past century, the exploration and identification of freshwater crabs in the Indian subcontinent have been highly productive, significantly contributing to global knowledge (Klaus et al. 2014). However, these efforts remain incomplete, primarily due to inadequate sampling and limited taxonomic research in this historically significant biogeographic zone (Balss 1957).

The Old World family Gecarcinucidae has a notable presence in India, with a total of 150 species documented worldwide (Mitra 2020; Pati et al. 2022; Mitra and Khamo 2024). Within the Indian subcontinent, 111 species spanning 30 genera have been documented, predominantly restricted to the peninsular region (Brandis and Sharma 2005; Bahir and Yeo 2007; Ng et al. 2008; Cumberlidge et al. 2009; Pati and Vargila 2019; Pati et al. 2019a, b; Mitra 2020; Raj et al. 2025). Some of these species primarily inhabit elevations exceeding 1000 meters within the Western Ghats, with some known solely from their type locality (Bahir and Yeo 2007). Due to the limited availability of occurrence data, most of these species are categorized as ‘Data Deficient’ on the IUCN Red List of Threatened Species (Cumberlidge et al. 2009). The Indian subcontinent also accommodates one of the taxonomically oldest genera, *Barytelphusa* Alcock, 1909, from which four additional genera—*Travancoriana* Bott, 1969, *Baratha* Bahir & Yeo, 2007, *Vanni* Bahir & Yeo, 2007, and *Vela* Bahir & Yeo, 2007—were later segregated based on a combination of morphological characters, including the presence of long flagella on the exopods of the third maxilliped and a relatively elongated distal segment of the second gonopod (G2). These genera are distinguished from other extant genera by their unique morphological traits and are primarily distributed in the Indian Peninsula (Bahir and Yeo 2007).

The genera *Vela* and *Baratha* are distinguished from *Vanni* and *Travancoriana* by their relatively convex carapace. The genus *Vela* is unique among its closely related species in possessing a distinctly convex carapace, a distinct groove at the suture between thoracic sternites 3 and 4, a slender male abdomen, and a telson that is 1.4 times longer than its proximal width. In contrast, *Baratha*, while sharing a convex carapace and a distinct groove at the suture between thoracic sternites 3 and 4, has a relatively broader male abdomen and a telson that is 1.1 times longer than its proximal width. The genus *Vanni*, characterized by a squarish and slightly convex carapace in frontal view, lacks a well-defined suture between thoracic sternites 3 and 4 and exhibits less distinct postorbital cristae, setting it apart from its related genera. Meanwhile, *Travancoriana* differs from all related genera by its low, transverse carapace, poorly marked suture between thoracic sternites 3 and 4, and strongly developed postorbital cristae. Despite these distinct morphological differences, molecular data for these genera remain scattered, complicating the understanding of gecarcinucid genetic diversity in peninsular India (Bahir and Yeo 2007).

In addition to rigorous taxonomic investigation, molecular phylogenetic analyses suggest that Gecarcinucidae originated within the Indian subcontinent during the Cretaceous/Early Paleogene period. This evolutionary insight is thought to be linked to the tectonic convergence of the Indian subcontinent with Eurasia. Hence, the systematic and phylogenetic studies on gecarcinucid crabs in the Indian subcontinent have gained significant momentum in recent years (Bossuyt et al. 2004; Bahir and Ng 2005; Bahir and Yeo 2005; Daniels et al. 2006; Klaus et al. 2006, 2009, 2010, 2013; Beenaerts et al. 2010). However, the continuous discovery of new gecarcinucid taxa underscores the incomplete understanding of their basal phylogenetic relationships. This necessitates further faunistic investigations to accurately assess their diversity and evolutionary history, particularly among montane crabs. This study is based on the hypothesis that additional, previously unreported gecarcinucid genera and species exist beyond their currently recognized biogeographic distribution in peninsular India, necessitating confirmation through an integrated approach. Thus, a recent zoological survey in the Eastern Ghats has led to the discovery of a new genus and a novel species within the family Gecarcinucidae.

## Material and methods

### Sampling and morphological examination

**Figure 1.**
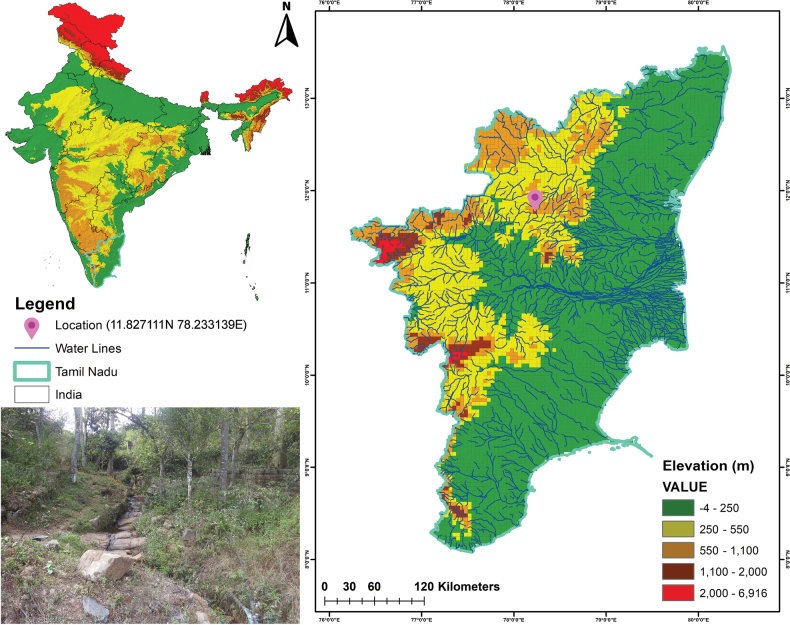
Map showing the collection and type locality of *Patithelphusa yercaudensis* gen. et sp. nov. An inset photo illustrates the habitat of this newly described taxon in the Eastern Ghats of India.

Freshwater crab specimens were hand-collected from a muddy microhabitat adjacent to a rocky stream in Manjakuttai, Shevaroy Hills, Yercaud, Salem District, Tamil Nadu, within the Eastern Ghats range (Fig. 1). Two whole-body specimens and body parts from four additional specimens were preserved in 70% ethanol. Morphological measurements and terminology followed previous studies (Ng 1988; Ng and Tay 2001; Guinot et al. 2013; Davie et al. 2015). Specimens were photographed using a Nikon P900 camera and examined under a Leica EZ4 stereozoom binocular microscope. The following abbreviations are used for morphological examination: CW = carapace width; CL = carapace length; FW = frontal width; CH = carapace height; P2–P5 = second to fifth ambulatory legs; S1–S8 = thoracic sternites 1–8; G1 = male first gonopod; G2 = male second gonopod; ZSIC = Zoological Survey of India, Crustacea Section; ZRC = Zoological Reference Collection, Lee Kong Chian Natural History Museum, Singapore.

### Comparative materials examined

*Baratha pushta* Bahir & Yeo, 2007: holotype male (cw 23.2 mm, cl 16.1 mm), ZRC 2003.0234; Loc. Vaguvurai estate, on Munnar-Pollachi, Kerala, India, 10°11'07.5"N, 77°06'40.0"E, alt. 1290 m — *Baratha peena* Bahir & Yeo, 2007: holotype male (cw 18.4 mm, cl 13.2 mm), ZRC 2003.0235; Paniyar estate, on Kumarly–Munnar road, Kerala, India, 10°00'47.0"N, 77°11'29.5"E, alt. 1260 m.

### DNA extraction, PCR amplification, and sequencing

The genomic DNA was extracted from the holotype and paratype specimens, as well as from body parts of four additional specimens using the standard phenol–chloroform isoamyl alcohol method (Sambrook and Russell 2001). The extracted DNA was visualized through 1% agarose gel electrophoresis. The published primer pair (16L29: 5′-YGCCTGTTTATCAAAAACAT-3′and 16H37: 5′-CCGGTYTGAACTCAAATCATGT3′) was used to amplify the widely applied partial 16S rRNA segment (⁓560 bp) for the identification of crab species (Klaus et al. 2009). The 25 ml PCR mixture comprises 10 pmol of each primer, 20 ng of DNA template, 1X PCR buffer, 1.0–1.5 mM of MgCl_2_, 0.25 mM of each dNTPs, and 1 U of Platinum Taq DNA Polymerase High fidelity (Invitrogen). The PCR reaction was performed in an Applied Biosystems MiniAmp Plus Thermal Cycler with the published thermal profile. The PCR products were purified using a QIAquick Gel Extraction Kit (Qiagen) according to the standard protocol. The cycle sequencing was executed by using a BigDye Terminator v. 3.1 Cycle Sequencing Kit (and 3.2 pmol of each primer on the Thermal Cycler). The products were cleaned using a BigDye X-terminator kit with standard protocol and subsequently bidirectionally sequenced by a ABI 3500 Genetic Analyzer (Applied Biosystems) at InBOL Healthcare Pvt Ltd, Kolkata, India.

### Sequence quality check and dataset preparation

The study obtained both forward and reverse chromatograms from the holotype and paratype samples. The noisy parts of each chromatogram were trimmed at both ends, and a quality value (> 40) was used to screen the consensus sequences through SeqScanner v. 1.0 (Applied Biosystems). The annotated sequences were verified through nucleotide BLAST search and subsequently contributed to GenBank for free public access. A total of 45 sequences of the gecarcinucids species distributed in India were retrieved from GenBank (Bossuyt et al. 2004; Daniels et al. 2006; Klaus et al. 2006; Shih et al. 2009; Beenaerts et al. 2010; Klaus et al. 2013; Klaus et al. 2014; Shashi et al. 2023; Wolfe et al. 2024) (Suppl. material 1: table S1). Additionally, nine sequences from two genera (*Pastilla* Ng & Tay, 2001 and *Mahatha* Ng & Tay, 2001) originating from Sri Lanka were incorporated into the dataset, as the region is geographically close to southern India (the actual study sites) and the faunal elements of both regions often exhibit close co-evolutionary affinities, including freshwater crabs (Bossuyt et al. 2004; Beenaerts et al. 2010). These sequences, along with the newly generated ones, were aligned using ClustalX to create a combined dataset for genetic distance and phylogenetic analyses. In addition, the DNA sequences of *Candidiopotamon penglai* (GenBank accession no. OR346860; family Potamidae) were included as an outgroup taxon in the present analyses (Shih et al. 2023).

### Genetic distance and phylogenetic analysis

The pairwise inter- and intra- group (species and genus) genetic distance was estimated through Kimura-2-parameter (K2P) in MEGA v. 11 (Tamura et al. 2021). The model selection (GTR+G+I) was conducted using JmodelTest v. 2, which yielded the lowest BIC (Bayesian Information Criterion) score (Darriba et al. 2012). Bayesian phylogenetic inference (BI) was conducted using MrBayes v. 3.1.2 (Ronquist and Huelsenbeck 2003), implementing a general time-reversible model with gamma-distributed rate variation and a proportion of invariable sites (nst = 6). The analysis employed four Metropolis-coupled Markov chain Monte Carlo (MCMC) chains, comprising one cold chain and three hot chains. A total of 1,000,000 generations were run, with tree sampling occurring every 100 generations, and the initial 25% of sampled trees were discarded as burn-in. Further, the maximum-likelihood (ML) phylogenetic tree was generated using the PhyML v. 3.0 web server (http://www.atgc-montpellier.fr/phyml/), incorporating 1000 bootstrap samples (Guindon et al. 2010). The final ML and BI phylogenies were visualized using the iTOL v. 4 web server (https://itol.embl.de/) (Letunic and Bork 2007).

## Results and discussion

### Taxonomic lineage

**Subphylum: Crustacea**

**Class: Malacostraca**

**Order: Decapoda**

**Family Gecarcinucidae Rathbun, 1904**

#### Patithelphusa gen. nov.

Taxon classificationAnimaliaDecapodaGecarcinucidae

D3A7991C-F3A1-5C88-81E6-977897B62DF1

https://zoobank.org/F7C2F210-A26D-4C76-8ED9-9E4CCB3AE1C6

##### Type species.

*Patithelphusa yercaudensis* sp. nov., by present designation.

##### Diagnosis.

**Figure 2.**
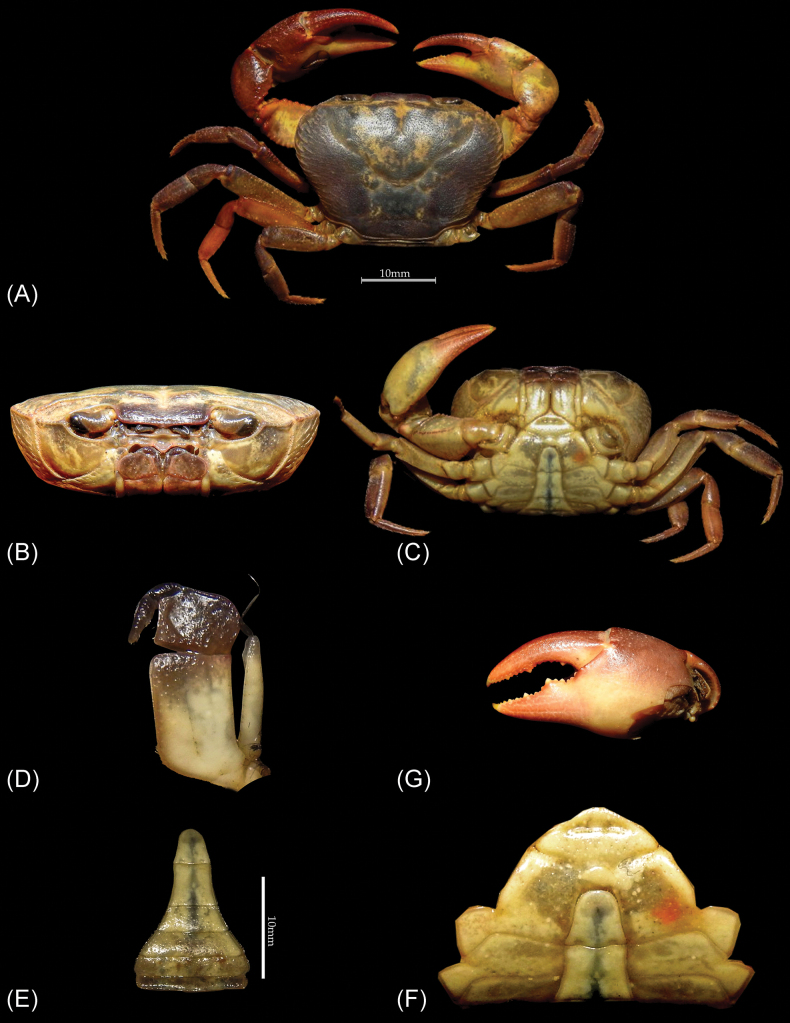
*Patithelphusa yercaudensis* sp. nov. holotype male (cw 29.22 mm, cl 20.83 mm) (ZSIC-CR 396): A. Dorsal view of the carapace; B. Frontal view of the cephalothorax; C. Ventral view of the body; D. Left third maxilliped with exopods; E. Pleon; F. Upper part of the sternum and pleon; G. Major cheliped hand.

**Figure 3.**
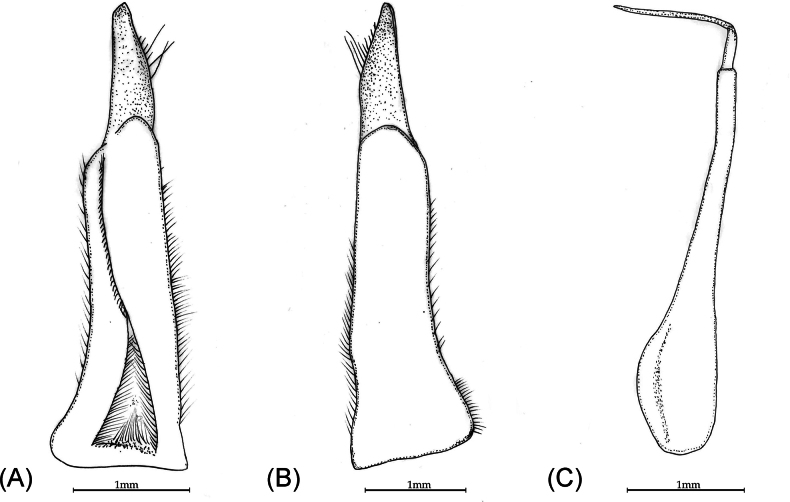
*Patithelphusa yercaudensis* sp. nov. holotype male (cw 29.22 mm, cl 20.83 mm) (ZSIC-CR 396). A. Ventral view of the left G1; B. Dorsal view of the left G1; C. Left lateral view.

Carapace distinctly broader than long, dorsal surface distinctly convex, lateral margins with many short oblique striae (Fig. 2A); dorsal surface distinctly convex in frontal view (Fig. 2B); external orbital angle widely triangular, blunt, outer margin serrated, 4.5 times of inner margin (Fig. 2A); epibranchial tooth short, distinct notch between epibranchial tooth and outer margin of external orbital tooth; cervical groove well demarcated in all course, broad and shallow anteriorly, relatively deep and narrow posteriorly, not reaching to the postorbital cristae; epistomal median lobe with distinct triangular tooth, tip bilobed; third maxilliped exopods with long flagellum (Fig. 2D); suture between S2/S3 shallow and broad, not reaching to lateral margins of sternum; suture between S3/S4 shallow and broad, not interrupted by any ridge, reaching to lateral edge of sternum. Sixth pleonal segment of male slightly broader than long (Fig. 2C, E, F); G1 subterminal segment relatively slender, basally broad; terminal segment cone shaped, elongated, 0.4 times of subterminal segment, inner margin straight, outer margin convex in middle, with some long setae, tip triangular, not sharp. G2 c. 1.2 times longer than G1, subterminal segment long, basally broad, terminal segment slightly shorter than half of subterminal segment (Fig. 3A–C).

##### Etymology.

The genus is named after eminent crustacean taxonomist, Dr Sameer Kumar Pati, for his valuable contribution to freshwater crab taxonomy in India. The gender is neuter.

##### Remarks.

The new genus *Patithelphusa* shares similarities with only four gecarcinucid genera, *Baratha*, *Travancoriana*, *Vanni* and *Vela*, by having a combination of characters: exopods of third maxilliped with long flagellum, and the distal segment of G2 is relatively long. *Patithelphusa* gen. nov. can nevertheless be separated from *Baratha* by its anterolateral and posterolateral region of carapace dorsal surface with many horizontal and oblique striae (Fig. 2A) (vs. anterolateral and posterolateral region of carapace dorsal surface smooth in *Baratha*; figs 19a, 21a, Bahir and Yeo 2007); external orbital angle widely triangular, blunt (Fig. 2A) (vs. external orbital angle sharp, produced; figs 19a, 21a, Bahir and Yeo 2007); pleonal somite six of male distinctly broader than long (Fig. 2C, E) (vs. pleonal somite six of male squarish to slight longer than broad; figs 18b, 19c, 21c, Bahir and Yeo 2007); antennular fossae rectangular (Fig. 2B) (vs. eye shaped, medially broad, gradually narrower in both ends, figs 19b, 21b, Bahir and Yeo 2007); G1 terminal segment relatively longer, tip blunt (Fig. 3A, B) (vs. G1 terminal segment relatively shorter, cone shaped, tip pointed; figs 18c–g, 20a–e, Bahir and Yeo 2007).

*Patithelphusa* gen. nov. can be separated from *Travancoriana* by several morphological characters: the dorsal surface of carapace distinctly convex in frontal and as well as in dorsal view in *Patithelphusa* gen. nov. (Fig. 2A, B) (vs. carapace dorsal surface slightly convex in frontal view in *Travancoriana*; taf. 4, figs 38, 39, Bott 1970; fig. 8a, b, Bahir and Yeo 2007); somite six of male pleon distinctly broader than long (Fig. 2C, E) (vs. somite six of male pleon longer than broad; taf. 4, fig. 40, Bott 1970; figs 7d, 8c, Bahir and Yeo 2007); outer margin of external orbital tooth serrated (Fig. 2A) (vs. outer margin of external orbital tooth smooth; taf. 4, fig. 39, Bott 1970; fig. 8a, Bahir and Yeo 2007); epigastric cristae separated from postorbital cristae by a small gap (Fig. 2A) (vs. epigastric cristae confluent with postorbital cristae; taf. 4, fig. 39, Bott 1970; fig. 8a, Bahir and Yeo 2007); epibranchial tooth small but discernible, cleft distinct (Fig. 2A) (vs. epibranchial tooth indistinct, cleft not visible; taf. 4, fig. 39, Bott 1970; fig. 8a, Bahir and Yeo 2007). Outer orbital margin of epibranchial tooth is distinctly straight in *Patithelphusa* (Fig. 2A) (vs. outer orbital margin of epibranchial tooth distinctly convex in *Travancoriana*; taf. 4, fig. 39, Bott 1970; fig. 8a, Bahir and Yeo 2007); G1 terminal segment cone shaped, elongated, 0.4 times of subterminal segment, tip triangular, not sharp (Fig. 3A, B) (vs. G1 terminal segment relatively longer c. 0.3-0.6 times of subterminal segment, tip acute; figs 7 f–h, 16 f-I, Bahir and Yeo 2007).

The genus *Vanni* superficially resembles *Patithelphusa* gen. nov., but the latter exhibits several distinguishing characteristics: carapace almost squarish in *Vanni* (fig. 32a, Bahir and Yeo 2007), while distinctly wider than long in *Patithelphusa* gen. nov. (Fig. 2A); carapace flat or slightly convex in frontal view (fig. 32b, Bahir and Yeo 2007), however the carapace of the new genus is convex in frontal and dorsal view (Fig. 2A, B); suture between S2/S3 indistinct or slightly visible in *Vanni* (fig. 32b, Bahir and Yeo 2007), while S2/S3 is distinctly visible in *Patithelphusa* gen. nov (Fig. 2C, F); suture between sternites S3/S4 indistinct (fig. 32b, Bahir and Yeo 2007) vs. S3/S4 distinct and reaches the lateral margins of the sternum in *Patithelphusa* gen. nov (Fig. 2C, F); G1 terminal segment cone shaped, elongated, 0.4 times of subterminal segment, inner margin straight, outer margin convex in middle, with some long setae; tip triangular, not sharp (Fig. 3A, B) (vs. G1 stout, terminal segment relatively short, c. 0.3-0.35 times of subterminal segment, subterminal segment gently tapering towards terminal segment; figs 31c–f, 33a, b, 35c–f, 33d–e, Bahir and Yeo 2007).

Genus *Vela* is closely related to the new genus, though there are some distinct morphological features that easily separate these two genera. The epigastric cristae is smooth in *Vela* (fig. 46a, Bahir and Yeo 2007), while it is distinctly rugose in *Patithelphusa* gen. nov. (Fig. 2A); postorbital cristae and epigastric cristae are confluent (fig. 46a, Bahir and Yeo 2007), however postorbital cristae and epigastric cristae are not confluent in *Patithelphusa* (Fig. 2A); sixth somite of the male pleon distinctly longer than broad (figs 45B, 46c, Bahir and Yeo 2007), however sixth somite broader than long in *Patithelphusa* (Fig. 2E); branchial region relatively swollen (fig. 46a, Bahir and Yeo 2007), while branchial region gently convex in *Patithelphusa* gen. nov. (Fig. 2A); G1 terminal segment cone shaped, 0.4 times of subterminal segment, inner margin straight, outer margin convex in middle (Fig. 3A, B) (vs. G1 terminal segment more slender and long, 0.45-0.5 times of subterminal segment, outer margin not convex; figs 45c–e, 47b–e: Bahir and Yeo 2007).

#### Patithelphusa yercaudensis sp. nov.

Taxon classificationAnimaliaDecapodaGecarcinucidae

ABF1BF47-A225-5133-9E57-2FEBCC6D0FAB

https://zoobank.org/F0FECF3D-2B9D-44A7-865C-01D11ACBA122

Figs 2, 4

##### Etymology.

This species is named after its type locality, Yercaud, a town and hill station in Salem District in Tamil Nadu, India. Located in the Shevaroy Hills in the Eastern Ghats, it is situated at an altitude of 1515 m. Used as a noun in apposition. Suggested common name: Yercaud crab.

##### Type specimens.

***Holotype***: • 1 male (cw 29.22 mm, cl 20.83 mm, ch 12.41 mm, fw 8.36 mm). Locality: Manjakuttai, Shevaroy Hills, Yercaud, District. Salem, Tamil Nadu, India, 11°49'37.6"N, 78°13'59.3"E; elevation 1504 m; date of collection, 27. 02. 2024; collected by S. Mitra; ZSIC Reg no. CR 396. ***Paratype***: • 1 female (cw 21.20 mm, cl 15.25 mm, ch 8.82 mm, fw 5.82 mm); collection data same as above; ZSIC Reg. no. CR 397.

##### Colourations.

Dark brown to chocolate colour in live condition. The cheliped and ventral colouration is light brown mixed with light yellow. The female is a little darker than the male.

##### Distribution.

The species is known only from the type locality, Yercaud (Fig. 1) of the District Salem, Tamil Nadu, India.

##### Diagnosis.

See genus Diagnosis.

##### Description.

Carapace distinctly broader than long (cw/cl =1.4), deep (ch/cl=0.6), dorsal surface gently convex in frontal view; epigastric cristae low, rugose, slightly anterior to postorbital cristae, postorbital cristae sharp extended up to epibranchial tooth; external orbital angle widely triangular, blunt, outer margin serrated, 4.5 times of inner margin; supraorbital margin sinuate, granulated (Fig. 2A), no notch between infraorbital margin and external orbital tooth (Fig. 2B); epibranchial tooth short, distinct notch between epibranchial tooth and outer margin of external orbital tooth; anterolateral margin distinctly convex, cristate, shorter than posterolateral margins, anterolateral region distinctly convex with some oblique striae, posterolateral margin gently concave.

Cervical groove well demarcated along its course, broad and shallow anteriorly, relatively deep and narrow posteriorly, not reaching to the postorbital cristae; mesogastric groove shallow, I-shaped; H-shaped groove distinct (Fig. 2A); sub-hepatic region rugose with some short striae, suborbital and pterygostomial region smooth. Front relatively narrow (fw/cw=0.3), orbit bluntly triangular, eyes occupy most of the orbital space, eye stalk massive, cornea large, frontal median triangle complete, very short; epistomal median lobe with a distinct triangular tooth, tip bilobed; antennae short, just reaching to the base of eyestalk; antennular fossae horizontally broad (Fig. 2B).

Third maxilliped ischium almost quadrangular, 1.6 times longer than broad, longitudinal median groove shallow, merus pentagonal, mesial margin straight; exopods longer than ischium, reaching third of merus, with long flagellum wider than merus (Fig. 2D). Suture between S1/S2 not visible, suture between S2/S3 as shallow broad groove not reaching to lateral margins of sternum; suture between S3/S4 shallow broad not interrupted by any ridge, reaching to lateral edge of sternum (Fig. 2C, F). Sternopleonal cavity reaching median part of the cheliped coxae, male pleon T-shaped, somite six squarish, proximal width slightly longer than median length, lateral margin gently concave; telson as long as broad, distinctly shorter than somite six, free end almost truncated to rounded (Fig. 2E).

Cheliped sub-equal, left cheliped slightly larger than right, major cheliped longer than P3, hands of major cheliped stout, cutting edge of fixed finger with large submedian teeth, along with 2–3 subteeth; cutting edge of movable finger with several small and 3–4 large triangular teeth, tips of both fingers pointed and curved; (Fig. 2G); ambulatory legs stout, merus of P3 shorter than carapace length.

G1 subterminal segment relatively slender, basally broad, terminal segment cone shaped, elongated, 0.4 times of subterminal segment, inner margin straight, outer margin convex in middle, with some long setae; tip triangular, not sharp (Fig. 3A, B). The G2 c. 1.2 times longer than G1, subterminal segment long, basally broad, terminal segment slightly shorter than half of the subterminal segment (Fig. 3C).

##### Variation.

**Figure 4.**
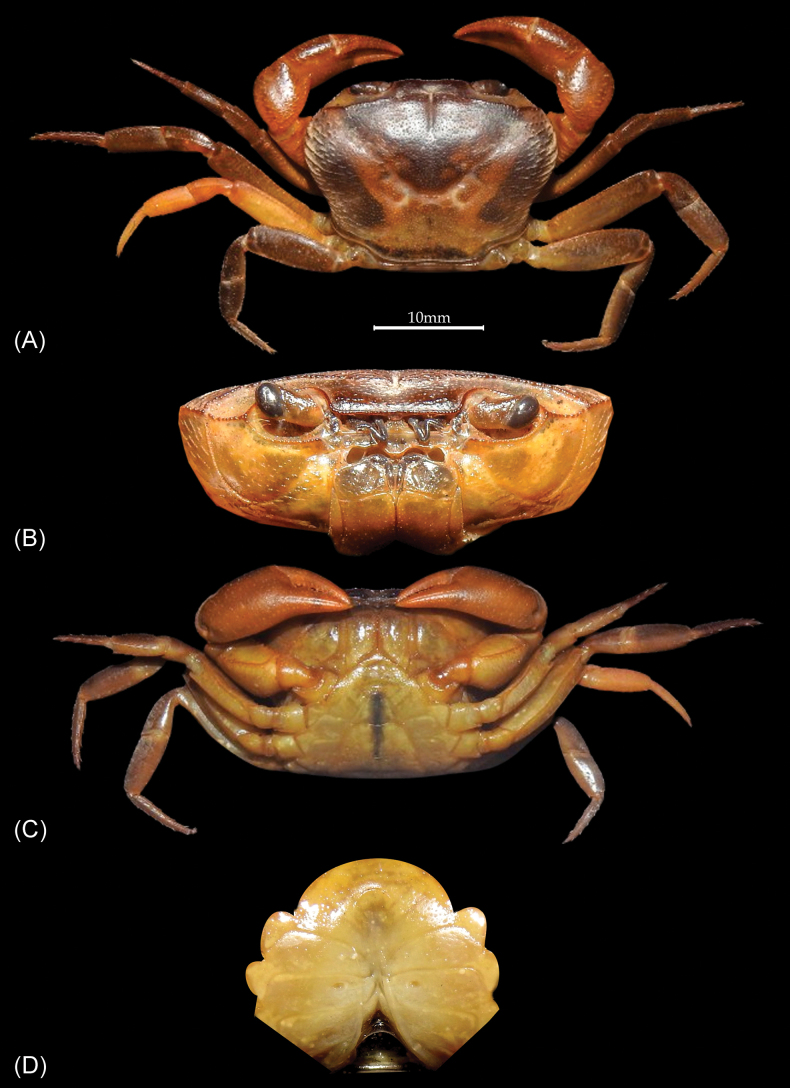
*Patithelphusa yercaudensis* sp. nov. paratype female (cw 21.20 mm, cl 15.25 mm) (ZSIC-CR 397). A. Dorsal view of the carapace; B. Frontal view of the cephalothorax; C. Ventral view; D. Gonopore and female sternum.

Paratype female is morphologically most similar to holotype male, except for the genital characters. Carapace gently convex, postorbital cristae prominent, cervical groove shallow broad, distinct along its course (Fig. 4A); female paratype, however, slightly differs in some morphological characters, median tooth of epistomal median lobe rounded, tip truncated (Fig. 4B) (vs. epistomal median lobe with triangular tooth, tip bilobed in holotype male, Fig. 2B); carapace less convex (Fig. 4A) (vs. carapace more convex in holotype male, Fig. 2A). Female specimen is not fully mature, hence female pleon not fully covering the sternum (Fig. 4C). Female gonopore small, horizontally oval, occupying approximately 1/5 of the length of S6, positioned close to S5/S6 (Fig. 4D).

##### Remarks.

The newly described species is currently the sole representative of the proposed monotypic genus *Patithelphusa*. This new species, *Patithelphusa yercaudensis* sp. nov., has some distinct morphological features; i.e., broad and shallow cervical groove, triangular bilobed median teeth on the epistomial median lobe, a blunt external orbital tooth with a relatively long outer margin and a distinct G1 terminal segment with a triangular tip (Figs 2A–F; 3A, B). In this study, the female paratype was observed to be smaller than the male holotype, raising the possibility of whether the differences reflect sexual dimorphism or ontogenetic variation. However, given the limited sample size, further investigation is recommended to confirm these morphological differences of the newly proposed species. Overall, the discovery of this new genus and species within Gecarcinucidae represents a significant contribution to the scientific understanding of freshwater crabs in India and globally. This novel species is meticulously described morphologically, with comparisons drawn to its closest relatives within the family Gecarcinucidae. With this addition, the total count of gecarcinucid crabs in India now stands at 112 species under 31 genera.

### Molecular identification and phylogenetic placement

**Figure 5.**
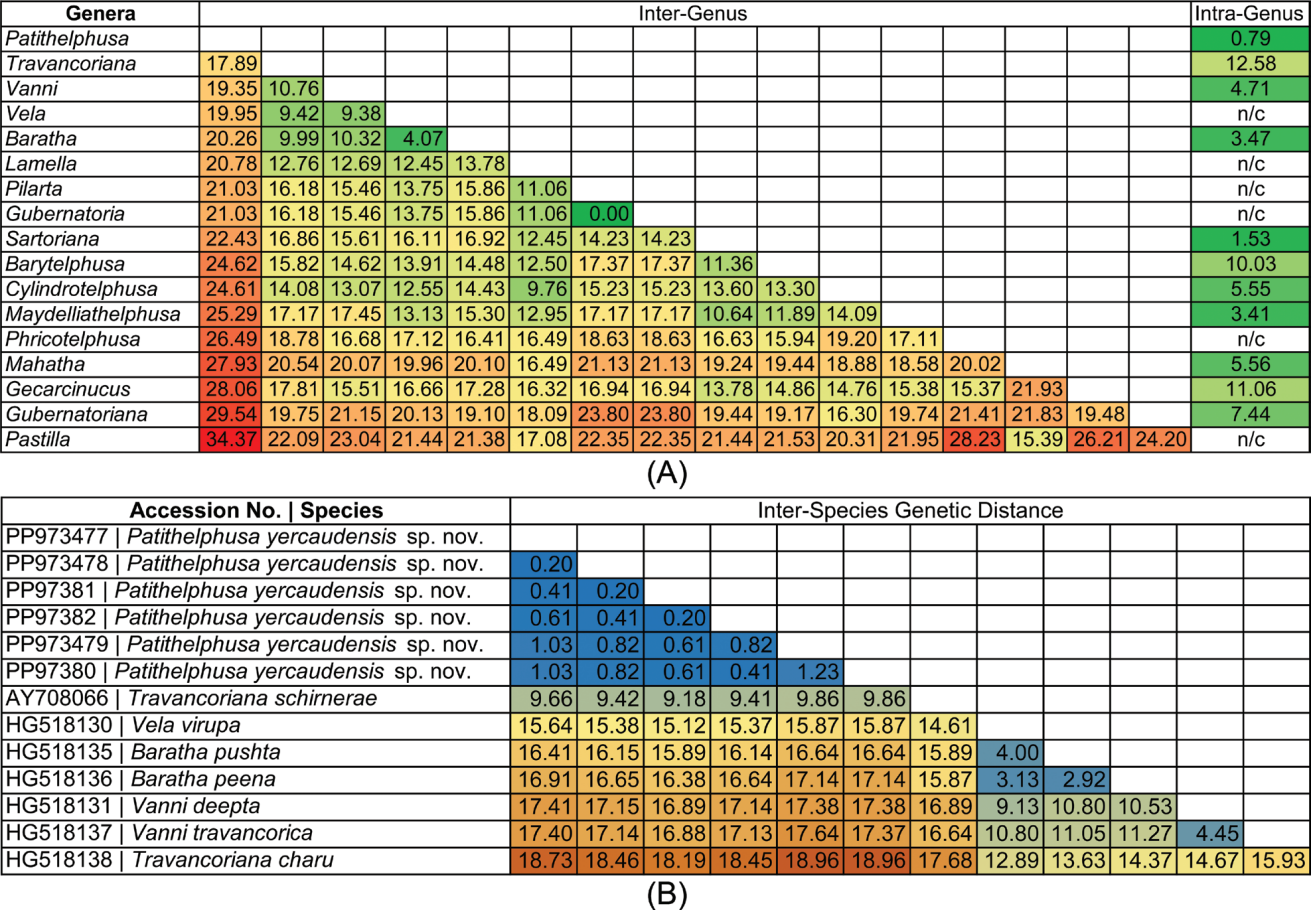
A. Heatmap illustrating the inter- and intra-rank genetic distances among various genera of the family Gecarcinucidae distributed across India and Sri Lanka; B. Heatmap depicting the interspecific genetic distances between the newly described species and morphologically similar species, based on the mitochondrial 16S rRNA gene.

**Figure 6.**
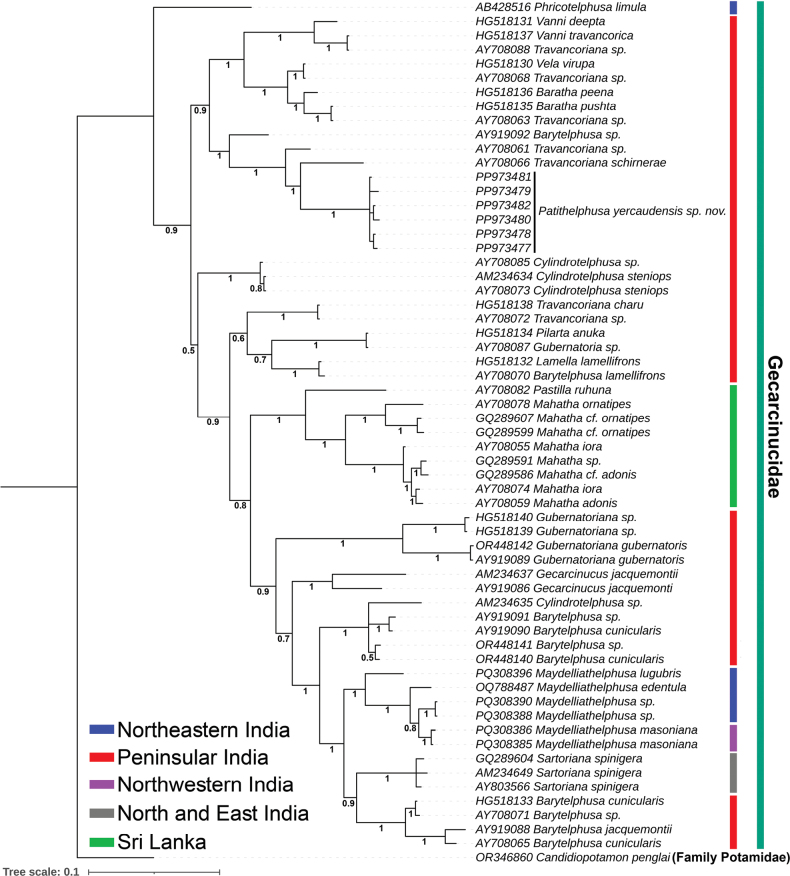
Bayesian phylogenetic analysis of the mitochondrial 16S rRNA gene revealed a distinct clustering of *Patithelphusa yercaudensis* sp. nov. compared to other Gecarcinucidae crab species. Black numbers represent posterior probability support values for each node. Distributions of Gecarcinucidae species are indicated by different colour bars adjacent to each clade.

The generated 16S rRNA gene sequences (Accession nos. PP973477 to PP973482) of the proposed new genus and species were submitted to GenBank. The newly proposed genus, *Patithelphusa* gen. nov., exhibits a high K2P genetic distance ranging from 17.89% (*Travancoriana*) to 34.37% (*Pastilla*) when compared with 17 recognized genera within the family Gecarcinucidae found in India and Sri Lanka (Fig. 5A). The proposed species, *Patithelphusa yercaudensis* sp. nov. also exhibited substantial genetic divergence from the morphologically similar lineages *Vela* (15.64%), *Baratha* (16.41%–16.91%), *Travancoriana* (9.66%–18.73%), and *Vanni* (17.40%–17.41%) (Fig. 5B). The newly described species also exhibits 0.20% to 1.23% intraspecific genetic divergence and shows closest genetic affinity with *Travancoriana schirnerae*, with a genetic distance of 9.66% (Fig. 5B). The phylogenetic analyses using both BI and ML approaches distinctly separated *Patithelphusa* gen. nov. and *P. yercaudensis* sp. nov. from other gecarcinucid taxa, with strong posterior probability and bootstrap support (Fig. 6, Suppl. material 1: fig. S1). Based on the clustering patterns observed, the proposed new genus and species were found to be closely allied with the genera *Travancoriana*, *Vanni*, *Vela*, and *Baratha*. The molecular analysis using partial mitochondrial 16S ribosomal RNA (rRNA) gene sequences supports the morphological hypothesis, clearly segregating the novel species from other known gecarcinucid taxa.

Notably, the genus *Travancoriana* exhibited non-monophyletic clustering and a high intra-generic genetic distance (12.58%), including two named species (*T. charu* Bahir & Yeo, 2007 and *T. schirnerae*) and five additional sequences identified only to the genus level (Fig. 5A). This pattern suggests the potential presence of multiple undescribed species within *Travancoriana*, warranting further taxonomic investigation. Overall, the phylogenetic reconstruction and genetic distance estimates presented in this study highlight the need for comprehensive taxonomic revisions within the Indian representatives of the family Gecarcinucidae. In particular, the findings underscore the potential for undocumented species diversity, especially within taxa distributed across the Indian subcontinent.

### Biogeographical inference

The evolutionary history and dispersal patterns of Old World gecarcinucid species have long intrigued carcinologists, particularly due to their diverse distribution and high degree of endemism. Several species are restricted to the mountainous regions of South Asia; however, molecular systematics evidence suggests that, in response to post-glacial sea-level rise, certain taxa have dispersed and successfully colonized islands across Southeast Asia (Klaus et al. 2009; Klaus et al. 2013). Notably, the Western Ghats, situated along the west coast of peninsular India, comprises a chain of isolated montane habitats or “sky islands” that support a remarkable assemblage of endemic taxa, including numerous species of gecarcinucid freshwater crabs (Bossuyt et al. 2004; Pati and Sharma 2014; Pati et al. 2016; Biju Kumar et al. 2017; Pati and Yeo 2022). Within this biogeographic complex, the distributional ranges of several genera, such as *Baratha*, *Travancoriana*, *Vanni*, and *Vela*, are either confined to their type localities or interrupted by major topographic features, such as the Palghat and Shencottah Gaps (Bott 1970; Bahir and Yeo 2007). In contrast, the Eastern Ghats, though a geologically older and more fragmented mountain range along the eastern margin of peninsular India, also displays significant faunal endemism, including a distinctive freshwater crab fauna (Mandal et al. 2022).

**Figure 7.**
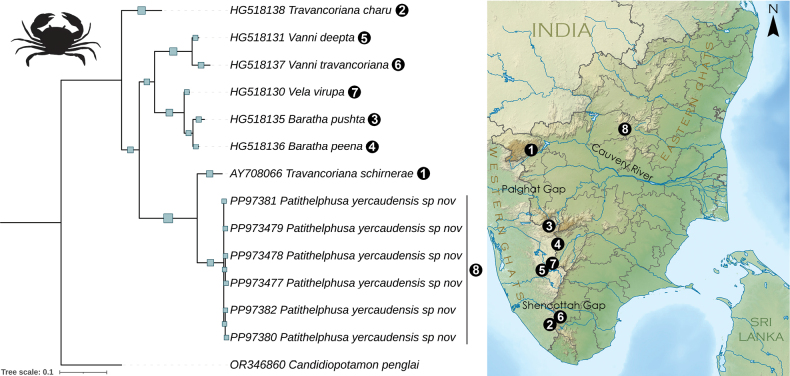
Pruned phylogeny illustrates the relationship of *Patithelphusa yercaudensis* sp. nov. with its morphologically close Gecarcinucidae species, alongside their known distributions in peninsular India. Locality data for *Baratha*, *Travancoriana*, *Vanni*, and *Vela* were derived from Bott (1970) and Bahir and Yeo (2007).

The present study reveals a monophyletic clade of the newly described species, phylogenetically distinct from its morphologically allied congeners (Fig. 7). This new lineage, described from the Shevaroy Hills in Yercaud, Eastern Ghats, shows a close phylogenetic affinity to *T. schirnerae*, a species originally reported from Coonoor and Hill Grove in the Nilgiris, located north of the Palghat Gap. Despite a relatively short aerial distance of 161 km between these localities, the intervening valleys and the Cauvery River system, with its complex tributary network, likely serve as ecological and geographical barriers restricting gene flow and limiting dispersal among these high-altitude, habitat-specialist decapods. This study also reveals a paraphyletic clustering within *Travancoriana* species, based on publicly available genetic data (*T. charu*: HG518138; *T. schirnerae*: AY708066), consistent with the previous study (Klaus et al. 2014). The substantial genetic divergence and non-monophyletic clustering of *Travancoriana* sequences in global databases suggest the existence of multiple cryptic species within the genus. These patterns highlight an exciting opportunity to expand our understanding of gecarcinucid diversity in the Western Ghats, suggesting that much of the region’s true species richness is yet to be discovered.

Thus, to gain deeper insights into the historical biogeography of gecarcinucid crabs across the Indian subcontinent, future research should prioritize extensive, fine-scale sampling throughout the Western and Eastern Ghats, particularly targeting lesser-explored hill ranges and ecological transition zones. The integrative taxonomic frameworks that combine detailed morphological analyses, multi-locus sequence data, and ecological information are expected to be pivotal for accurately delineating species boundaries, uncovering cryptic diversity, and elucidating the evolutionary processes underlying speciation and endemism in montane freshwater crab lineages (Schubart and Ng 2008; Cumberlidge et al. 2009; Tsang et al. 2014; Daniels et al. 2023; Wolfe et al. 2024). These multidisciplinary approaches are vital not only for refining the taxonomic resolution of gecarcinucid crabs but also for identifying microendemic populations and evolutionarily significant units, thereby providing a scientific basis for targeted conservation planning and sustainable habitat management in the ecologically sensitive and increasingly threatened landscapes of both Western and Eastern Ghats in India.

## Supplementary Material

XML Treatment for Patithelphusa

XML Treatment for Patithelphusa yercaudensis

## ﻿Additional information

### Use of AI

No use of AI was reported.

### Author ORCIDs

Santanu Mitra https://orcid.org/0000-0002-3247-9293

Shibananda Rath https://orcid.org/0000-0002-3547-3743

Won-Kyo Jung https://orcid.org/0000-0002-1615-750X

Hyun-Woo Kim https://orcid.org/0000-0003-1357-5893

Shantanu Kundu https://orcid.org/0000-0002-5488-4433

### Data availability

The examined specimens are vouchered in the National Zoological Collections of the Crustacea Division, Zoological Survey of India, and can be re-examined upon obtaining the necessary permissions from the authors and the Director Zoological Survey of India, Kolkata. The DNA sequences generated in this study (Accession numbers PP973477–PP973482) have been submitted to the global NCBI GenBank database (https://www.ncbi.nlm.nih.gov/) and are freely accessible for future research.

## Supplementary materials

Supplementary material 1 Supplementary information Data type doc Explanation note **fig. S1.** Maximum-likelihood phylogenetic analysis of the mitochondrial 16S rRNA gene revealed the evolutionary relationships between *Patithelphusa yercaudensis* sp. nov. and other species within the family Gecarcinucidae. Bootstrap support of each node is indicated numerically. **table S1.** The dataset used in the present study was prepared using both the generated DNA sequences and sequences retrieved from the GenBank database. https://binary.pensoft.net/file/1452983This dataset is made available under the Open Database License (http://opendatacommons.org/licenses/odbl/1.0/). The Open Database License (ODbL) is a license agreement intended to allow users to freely share, modify, and use this Dataset while maintaining this same freedom for others, provided that the original source and author(s) are credited.Santanu Mitra, Shibananda Rath, Won-Kyo Jung, Hyun-Woo Kim, Shantanu Kundu
