# Supplementary material for: Scuttling in the highlands: Discovery of a new genus and species of freshwater crabs (Decapoda, Gecarcinucidae) from the Eastern Ghats, India
Source: Zookeys. 2025 Oct 28;1257:71–90. doi: 10.3897/zookeys.1257.156494 (PMC12587172; doi:10.3897/zookeys.1257.156494)
Supplement: Supplementary material 1 — Supplementary information [file zookeys-1257-071_article-156494__-s001.doc]

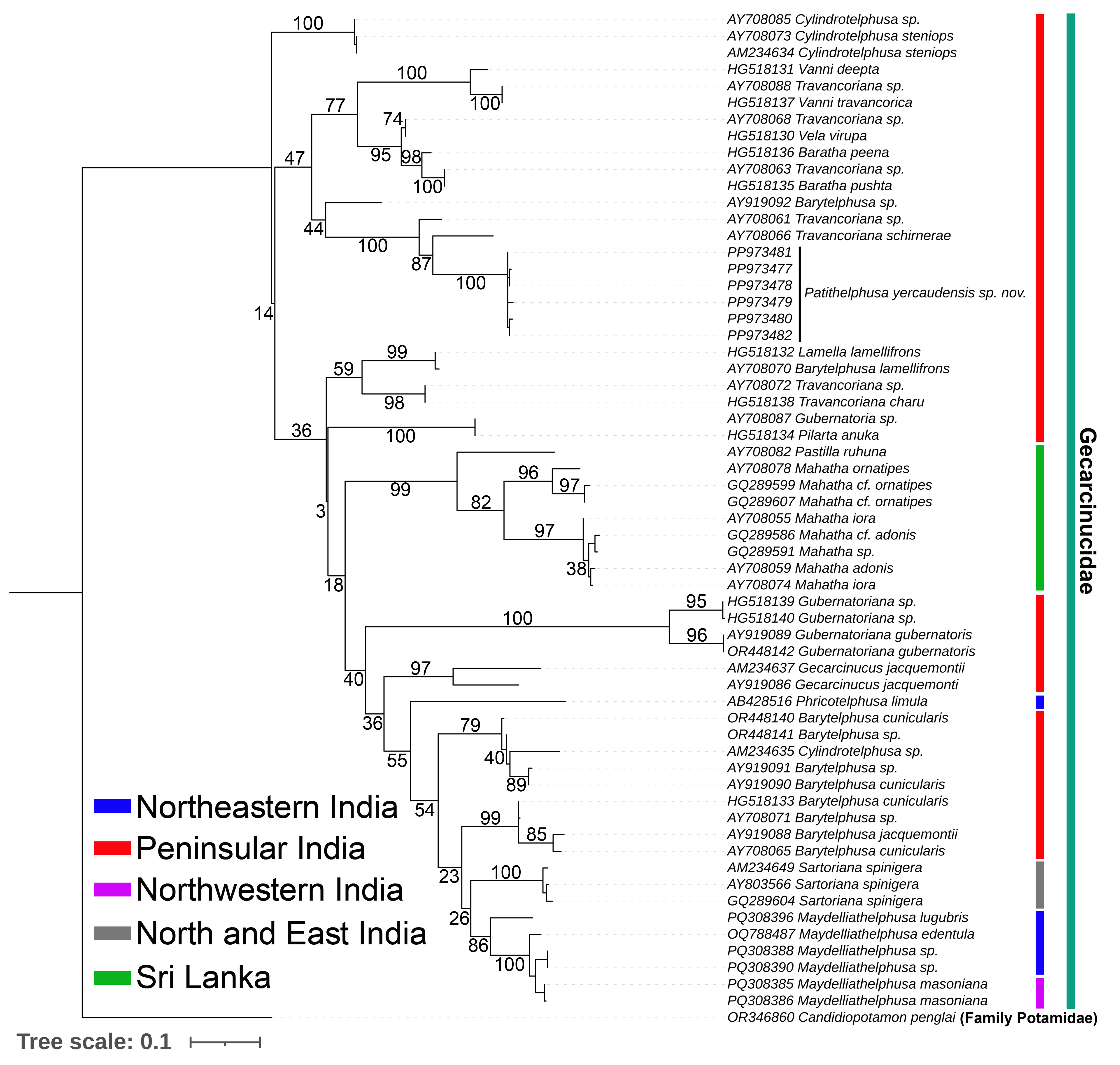
**Supplementary Materials**

**Figure S1.** Maximum-Likelihood phylogenetic analysis of the mitochondrial 16S rRNA gene revealed the evolutionary relationships between *Patithelphusa yercaudensis* sp. nov. and other species within the family Gecarcinucidae. Bootstrap support of each node indicated numerically.

**Table S1.** The dataset used in the present study was prepared using both the generated DNA sequences and sequences retrieved from the GenBank database.

| **Sl. No.** | **Family** | **Accession No.** | **Species** | **References** |
| --- | --- | --- | --- | --- |
| 1 | Gecarcinucidae | PP973477 | *Patithelphusa_yercaudensis_*sp._nov. | This Study |
| 2 | Gecarcinucidae | PP973478 | *Patithelphusa_yercaudensis_*sp._nov. | This Study |
| 3 | Gecarcinucidae | PP973479 | *Patithelphusa_yercaudensis_*sp._nov. | This Study |
| 4 | Gecarcinucidae | PP973480 | *Patithelphusa_yercaudensis_*sp._nov. | This Study |
| 5 | Gecarcinucidae | PP973481 | *Patithelphusa_yercaudensis_*sp._nov. | This Study |
| 6 | Gecarcinucidae | PP973482 | *Patithelphusa_yercaudensis_*sp._nov. | This Study |
| 7 | Gecarcinucidae | AY708072 | *Travancoriana_*sp. | Bossuyt et al. 2004 |
| 8 | Gecarcinucidae | AY708063 | *Travancoriana_*sp. | Bossuyt et al. 2004 |
| 9 | Gecarcinucidae | AY708061 | *Travancoriana_*sp. | Bossuyt et al. 2004 |
| 10 | Gecarcinucidae | AY708068 | *Travancoriana_*sp. | Bossuyt et al. 2004 |
| 11 | Gecarcinucidae | AY708088 | *Travancoriana_*sp. | Bossuyt et al. 2004 |
| 12 | Gecarcinucidae | HG518138 | *Travancoriana_charu* | Klaus et al. 2014 |
| 13 | Gecarcinucidae | AY708066 | *Travancoriana_schirnerae* | Bossuyt et al. 2004 |
| 14 | Gecarcinucidae | HG518137 | *Vanni_travancoriana* | Klaus et al. 2014 |
| 15 | Gecarcinucidae | HG518131 | *Vanni_deepta* | Klaus et al. 2014 |
| 16 | Gecarcinucidae | HG518130 | *Vela_virupa* | Klaus et al. 2014 |
| 17 | Gecarcinucidae | HG518136 | *Baratha_peena* | Klaus et al. 2014 |
| 18 | Gecarcinucidae | HG518135 | *Baratha_pushta* | Klaus et al. 2014 |
| 19 | Gecarcinucidae | OR448142 | *Gubernatoriana_gubernatoris* | Wolfe et al. 2024 |
| 20 | Gecarcinucidae | AY919089 | *Gubernatoriana_gubernatoris* | Daniels et al. 2006 |
| 21 | Gecarcinucidae | HG518140 | *Gubernatoriana_*sp. | Klaus et al. 2014 |
| 22 | Gecarcinucidae | HG518139 | *Gubernatoriana_*sp. | Klaus et al. 2014 |
| 23 | Gecarcinucidae | AY708087 | *Gubernatoria* sp. | Bossuyt et al. 2004 |
| 24 | Gecarcinucidae | OR448140 | *Barytelphusa_cunicularis* | Wolfe et al. 2024 |
| 25 | Gecarcinucidae | HG518133 | *Barytelphusa_cunicularis* | Klaus et al. 2014 |
| 26 | Gecarcinucidae | AY708065 | *Barytelphusa_cunicularis* | Bossuyt et al. 2004 |
| 27 | Gecarcinucidae | AY919090 | *Barytelphusa_cunicularis* | Daniels et al. 2006 |
| 28 | Gecarcinucidae | AY708070 | *Barytelphusa_lamellifrons* | Bossuyt et al. 2004 |
| 29 | Gecarcinucidae | AY919088 | *Barytelphusa_jacquemontii* | Daniels et al. 2006 |
| 30 | Gecarcinucidae | OR448141 | *Barytelphusa_*sp. | Wolfe et al. 2024 |
| 31 | Gecarcinucidae | AY708071 | *Barytelphusa_*sp. | Bossuyt et al. 2004 |
| 32 | Gecarcinucidae | AY919092 | *Barytelphusa_*sp. | Daniels et al. 2006 |
| 33 | Gecarcinucidae | AY919091 | *Barytelphusa_*sp. | Daniels et al. 2006 |
| 34 | Gecarcinucidae | HG518134 | *Pilarta_anuka* | Klaus et al. 2014 |
| 35 | Gecarcinucidae | HG518132 | *Lamella_lamellifrons* | Klaus et al. 2014 |
| 36 | Gecarcinucidae | AY708073 | *Cylindrotelphusa_steniops* | Bossuyt et al. 2004 |
| 37 | Gecarcinucidae | AM234634 | *Cylindrotelphusa_steniops* | Klaus et al. 2006 |
| 38 | Gecarcinucidae | AY708085 | *Cylindrotelphusa* sp. | Bossuyt et al. 2004 |
| 39 | Gecarcinucidae | AM234635 | *Cylindrotelphusa_*sp. | Klaus et al. 2006 |
| 40 | Gecarcinucidae | AY919086 | *Gecarcinucus_jacquemonti* | Daniels et al. 2006 |
| 41 | Gecarcinucidae | AM234637 | *Gecarcinucus_jacquemontii* | Klaus et al. 2006 |
| 42 | Gecarcinucidae | AY803566 | *Sartoriana_spinigera* | Daniels et al. 2006 |
| 43 | Gecarcinucidae | GQ289604 | *Sartoriana_spinigera* | Beenaerts et al. 2010 |
| 44 | Gecarcinucidae | AM234649 | *Sartoriana_spinigera* | Klaus et al. 2006 |
| 45 | Gecarcinucidae | PQ308390 | *Maydelliathelphusa_*sp. | Unpublished |
| 46 | Gecarcinucidae | PQ308388 | *Maydelliathelphusa_*sp. | Unpublished |
| 47 | Gecarcinucidae | PQ308386 | *Maydelliathelphusa_masoniana* | Unpublished |
| 48 | Gecarcinucidae | PQ308385 | *Maydelliathelphusa_masoniana* | Unpublished |
| 49 | Gecarcinucidae | OQ788487 | *Maydelliathelphusa_edentula* | Shashi et al. 2023 |
| 50 | Gecarcinucidae | PQ308396 | *Maydelliathelphusa_lugubris* | Unpublished |
| 51 | Gecarcinucidae | AB428516 | *Phricotelphusa_limula* | Shih et al. 2009 |
| 52 | Gecarcinucidae | AY708082 | *Pastilla_ruhuna* | Bossuyt et al. 2004 |
| 53 | Gecarcinucidae | AY708078 | *Mahatha_ornatipes* | Bossuyt et al. 2004 |
| 54 | Gecarcinucidae | GQ289607 | *Mahatha* cf. *ornatipes* | Beenaerts et al. 2010 |
| 55 | Gecarcinucidae | GQ289599 | *Mahatha_*cf. *ornatipes* | Beenaerts et al. 2010 |
| 56 | Gecarcinucidae | AY708074 | *Mahatha_iora* | Bossuyt et al. 2004 |
| 57 | Gecarcinucidae | AY708055 | *Mahatha_iora* | Bossuyt et al. 2004 |
| 58 | Gecarcinucidae | AY708059 | *Mahatha_adonis* | Bossuyt et al. 2004 |
| 59 | Gecarcinucidae | GQ289586 | *Mahatha* cf. *adonis* | Beenaerts et al. 2010 |
| 60 | Gecarcinucidae | GQ289591 | *Mahatha_*sp. | Beenaerts et al. 2010 |
| 61 | Potamidae | OR346860 | *Candidiopotamon_penglai* | Shih et al. 2023 |
